# Eye Development in *Sepia officinalis* Embryo: What the Uncommon Gene Expression Profiles Tell Us about Eye Evolution

**DOI:** 10.3389/fphys.2017.00613

**Published:** 2017-08-24

**Authors:** Boudjema Imarazene, Aude Andouche, Yann Bassaglia, Pascal-Jean Lopez, Laure Bonnaud-Ponticelli

**Affiliations:** ^1^UMR Biologie des Organismes et Ecosystèmes Aquatiques, Museum National d'Histoire Naturelle, Sorbonne Universités, Centre National de la Recherche Scientifique (CNRS 7208), Université Pierre et Marie Curie (UPMC), Université de Caen Normandie, Institut de Recherche Pour le Développement (IRD207), Université des Antilles Paris, France; ^2^Université Paris Est Créteil-Val de Marne Paris, France

**Keywords:** eye development, *Sepia officinalis*, *dac*, *six*, *eya*, *rhodopsin*

## Abstract

In metazoans, there is a remarkable diversity of photosensitive structures; their shapes, physiology, optical properties, and development are different. To approach the evolution of photosensitive structures and visual function, cephalopods are particularly interesting organisms due to their most highly centralized nervous system and their camerular eyes which constitute a convergence with those of vertebrates. The eye morphogenesis in numerous metazoans is controlled mainly by a conserved Retinal Determination Gene Network (RDGN) including *pax, six, eya*, and *dac* playing also key developmental roles in non-retinal structures and tissues of vertebrates and *Drosophila*. Here we have identified and explored the role of *Sof-dac, Sof-six1/2, Sof-eya* in eye morphogenesis, and nervous structures controlling the visual function in *Sepia officinalis*. We compare that with the already shown expressions in eye development of *Sof-otx* and *Sof-pax* genes. *Rhodopsin* is the pigment responsible for light sensitivity in metazoan, which correlate to correlate visual function and eye development. We studied *Sof-rhodopsin* expression during retina differentiation. By *in situ* hybridization, we show that (1) all of the RDGN genes, including *Sof-pax6*, are expressed in the eye area during the early developmental stages but they are not expressed in the retina, unlike *Sof-otx*, which could have a role in retina differentiation; (2) *Sof-rhodopsin* is expressed in the retina just before vision gets functional, from stage 23 to hatching. Our results evidence a role of *Sof-six1/2, Sof-eya*, and *Sof-dac* in eye development. However, the gene network involved in the retinal photoreceptor differentiation remains to be determined. Moreover, for the first time, *Sof-rhodopsin* expression is shown in the embryonic retina of cuttlefish suggesting the evolutionary conservation of the role of *rhodopsin* in visual phototransduction within metazoans. These findings are correlated with the physiological and behavioral observations suggesting that *S. officinalis* is able to react to light stimuli from stage 25 of organogenesis on, as soon as the first retinal pigments appear.

## Introduction

In metazoans, the evolution of photosensitives structures is difficult to establish as there are a high diversity of shapes, at histological and cellular level, and functioning at physiological and optical level, and analogous “eyes” appeared during evolution several times in different lineages (Land, 1988). Nevertheless, “eye” morphogenesis is controlled by a conserved genetic network of transcription factors (Gehring, 2002). Among these genes, *pax6* is a member of the highly conserved paired-box family of transcription factors (Burri et al., 1989; Noll, 1993). *Pax6* is considered as a universal master gene controlling eye morphogenesis, and its expression is reported in developing photoreceptors (Echelard et al., 1993; Chi and Epstein, 2002; Pichaud and Desplan, 2002; Gehring, 2005; for review see Kumar, 2009).

In vertebrates as in *Drosophila*, genes that govern eye specification are numerous. Indeed, eye formation is known to be controlled particularly by the Retinal Determination Gene Network (RDGN). It includes *pax6, eya (eyes absent), six (sine oculis*), and *dac (dachshund)* which act as a regulatory network of eye formation and retinal differentiation (Kumar and Moses, 2001; Donner and Maas, 2004). More studies indicate that these genes are also involved in the proliferation of progenitor cells, differentiation of retinal precursors, specification and/or maintenance of photoreceptor neurons and finally in the development of many other non-retinal tissues and organs (Bessa et al., 2002; Brodbeck and Englert, 2004; Christensen et al., 2008; Lopes and Casares, 2009; Peng et al., 2009). *Pax6, six3, six6, eya1, eya2, eya3*, and *Dach1* are known to play crucial roles in eye development in vertebrates. Furthermore, it has been shown that *pax6* is an upstream regulator in the RDGN in *Drosophila* (Czerny et al., 1999). Besides this network, *otx (Orthodenticle homeobox 2)* and *Notch* play a key role in photoreceptor cell differentiation and retinal organization (for review see Boyl et al., 2001; Buresi et al., 2012; Koenig et al., 2016). *In fine*, photoreception is allowed by the presence of pigments of the *opsin* family, present in all groups whatever the structure of the photoreceptor cells (Gehring, 2002). *Opsin* proteins are known to be involved both in visual and extraocular phototransduction (Porter et al., 2011). The signal cascade of visual phototransduction is initiated in the retinal photoreceptors when a photon is absorbed by a G protein-coupled receptor that is attached to a vitamin A-derived chromophore, 11-cis-retinal. The activated visual pigment molecule (*opsin*) induces a transduction cascade that results in the opening or closing of cation cGMP-gated channels in the photoreceptors (Hargrave, 2001).

Among metazoans, researchers are beginning to study the RDGN in lophotrochozoans. It has been shown that this regulatory network is involved in morphogenesis of the pigment-cup eyes of *Terebratalia transversa* (Passamaneck et al., 2011) and *Platynereis dumerilii* (Arendt et al., 2002), of the eyespot of *Lineus sanguineus* and *Leptochiton asellus* (Loosli et al., 1996; Vöcking et al., 2015), and of the cup eye of *Dugesia japonica* (Dong et al., 2012; Kamijyo et al., 2015). Within lophotrochozoans, cephalopods are good model species in the context of research of evolution and development (Evo-Devo) due to their highly centralized nervous system that is more centralized than in any group of invertebrates (Zullo and Hochner, 2011) and their specific “complex” camerular eyes, which constitute a convergence with those of vertebrates.

**Figure 1:**
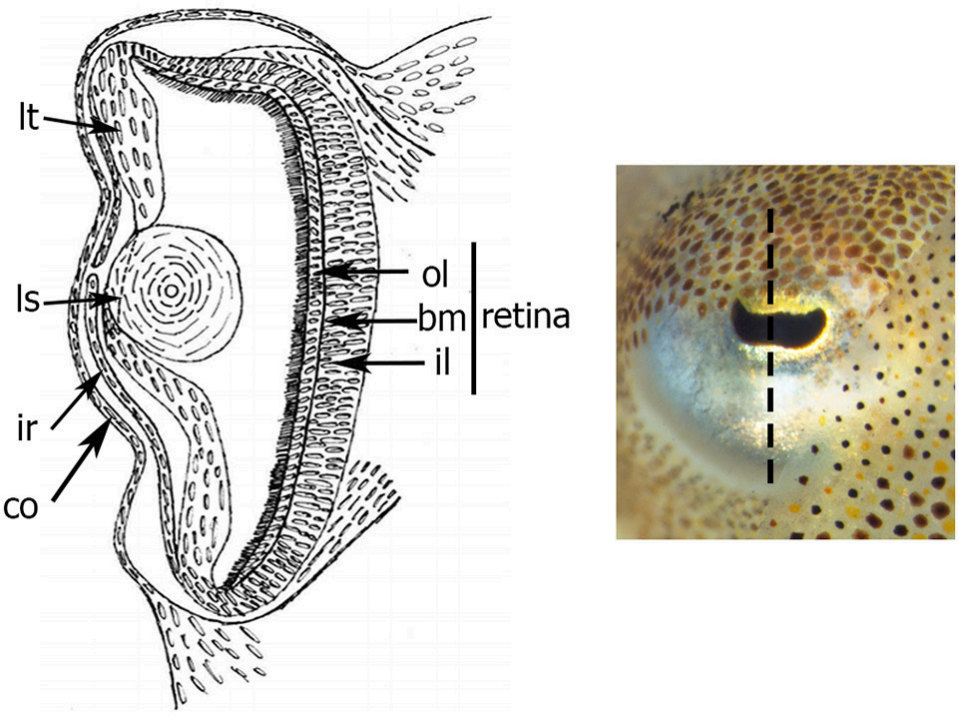
Schema of transversal section of the eye of *S. officinalis* at stage 30. Black dotted-line shows the level and the orientation of the section. bm: basement membrane; co: cornea; il: inner layer; ir: iris; ls: lens; lt: lentigenic tissue; ol: outer layer.

The cephalopod eye consists from the inside to the outside of: a retina covering the deepest part of the optic vesicle, a lens “closing” the vesicle, an iris and a cornea covering the eye (Figure 1). The retina is composed of rhabdomeric photoreceptor cells supported by a layer of support cells. Each photoreceptor consists of an outer (posterior) segment containing the nuclei and an inner (anterior) segment. The two segments are limited by a basement membrane. The development of the eye has been described in *Sepiella japonica*, in *Sepioteuthis australis*, in *Loligo vulgaris*, and recently in *Doryteuthis pealeii* (Marthy, 1973; Yamamoto, 1985; Bozzano et al., 2009; Koenig et al., 2016). The iris and cornea derive from two layers (respectively inner and outer) of ectodermal and mesodermal tissues growing around the optic vesicle (Lemaire and Richard, 1978; Tomarev et al., 1997); the circular lens is produced by lentigenic cells (West et al., 1995), and the retina, is formed during invagination of the primary optic vesicle (Lemaire, 1971; Lemaire and Richard, 1978).

Studies about the cephalopod's photosensitivity during embryogenesis have suggested that embryos become photosensitive early before hatching before the final differentiation of the retina (*S. japonica*, Yamamoto et al., 1985; *S. australis*, Bozzano et al., 2009). Unlike any other cephalopod, *Sepia officinalis* embryos develop in a dark visual environment because of the black capsule surrounding the egg, which attenuates the light reaching the embryo. Nevertheless, *S. officinalis* is able to react to light stimulus from stage 25 of organogenesis, i.e., as soon as the first retinal pigments appear (Lemaire, 1971; Lemaire and Richard, 1978; Romagny et al., 2012). Maturation of the visual system occurs in the last stages of photoreceptor differentiation before hatching: the main elements for photosensitive function and the cuttlefish's eyes are entirely functional at hatching, as the juvenile immediately adopts the visual-guided behavior of predation. It must be noted that cephalopods are known to have a remarkable capacity to transform their appearance by changing their dermal coloration, patterning and shape using chromatophores, iridophores, leucophores, and papillae (Cloney and Brocco, 1983; Allen et al., 2009). The skin pattern is controlled by the eye and probably by an extraocular or non-visual photoreception, as shown in some cephalopod species (Kingston et al., 2015a). All of these extraocular photoreceptors described are known to use many phototransduction components including *retinochrome, visual arrestin, rhodopsin kinase*, and *rhodopsin* identical to the isoform expressed in the eyes (Tong et al., 2009).

The development of the cephalopod eye is investigated in numerous molecular and genomic studies (Tomarev et al., 1997; Hartmann et al., 2003; Bozzano et al., 2009; Ogura et al., 2013; Peyer et al., 2014; Yoshida et al., 2014; Koenig et al., 2016). To complete the behavioral approaches and to understand mechanisms of eye maturation and visual function appearance, we have chosen to explore the molecular pathways underlying the developmental processes in an evolutionary perspective in *S. officinalis. Pax6* expression has been already determined in numerous cephalopods. During development, *pax6* is expressed in eyes particularly in the ocular primordia, optic ganglia, and light organ (a photosensitive structure of bobtail squid) (Tomarev et al., 1997: *Loligo opalescens*; Hartmann et al., 2003: *Euprymna scolopes*; Navet et al., 2009: *S. officinalis*; Peyer et al., 2014: *E. scolopes*; Yoshida et al., 2014: *Idiosepius paradoxus*; Koenig et al., 2016: *D. pealeii*). Expression of other genes of the RDGN, *six, eya*, and *dac* has been described in the central nervous system, optic area and light organ of *E. scolopes* and during eye morphogenesis of the *D. pealeii* embryo (Peyer et al., 2014; Koenig et al., 2016). Finally, *Sof-otx* expression has been characterized in *S. officinalis* embryo in early to late organogenesis of the eye (Buresi et al., 2012). Nonetheless, most of these studies have been performed on wholemount embryo, without considering specifically the retina morphogenesis and its function.

Our goal was to understand the evolutionary mechanisms, the complexity and the emergence of photosensitive structures and visual phototransduction in a cephalopod group. Thus, we described the morphological differentiation of the retina, and complement the identification and description by spatio-temporal expression patterns of the *Sof-six1/2, Sof-eya, Sof*-*dac* genes. Then, we highlight and discuss the role of these genes during eye specification in *S. officinalis* including the *pax genes* and *otx* expressions in the eyes. Furthermore, in order to link the RDG network with the visual phototransduction components and the appearance of photosensitivity during the development, *Sof-rhodopsin* expression patterns were explored in the developing retina.

## Materials and methods

### Collection, *S. officinalis* eggs incubation, and staging

In France, cuttlefish experiment and maintenance are covered under European Union guidelines (Directive 86/609) and the French law (decree 87/848) regulating animal experimentation that does not concerns embryos before hatching. Nonetheless, all experiments were performed according to France and European ethical guidelines in the treatment and handling of all animals used within this study. Fertilized eggs of *S. officinalis* used in this study come from SMEL (Synergie MEr et Littoral, Blainville, France). The protocols for the staging and the fixation of the embryos are detailed in Buresi et al. (2012).

### Phylogenetic analysis, characterization, and sequencing of *S. officinalis* genes

mRNA fragments of *Sof-six1/2, Sof-eya, Sof-dac*, and *Sof-opsin* were characterized in an embryonic EST library of *S. officinalis* (ADY0AAA48YE16CM1, tc_01401, ADY0AAA73Y015CM1, and tc_01048 respectively; Bassaglia et al., 2012). For the phylogenetic analyzes all alignments were performed using MAFFT and the G-INS-I iterative refinement method (Katoh and Standley, 2013). The best maximum-likelihood trees were inferred using PhyML with the WAG evolutionary model and 100 bootstrap replicates. Each of the genes has been tested in a phylogenetic work including genes from other metazoans. Six is identified as a six1/2, and our *opsin* sequence shows exactly the same sequence as that of AY450853 and that of O16005, identified as a true Gq-coupled/rhabdomeric photoreceptor *opsin* in the phylogenetic tree of Yoshida et al. (2015). From these characterized sequences, specific primers were designed for PCR amplification: *Sof-rhodopsinF3* (5′-GTACAACCCCACCATGGAGG-3′) and *Sof-rhodopsinR3* (5′-CGCCGATGAAGCCGTATACT-3′), *Sof-six1/2F3* (5′-CCTCCCATGCTTCCATCGTT-3′) and *Sof-six1/2R3* (5′-GAAATTTTCGGCGGACCCTG-3′), *Sof-eyaF1* (5′-ACCTACACGAGGTGGTCGTC-3′), and *Sof-eyaR1* (5′-CCACGGACTCCAGTTGCTAT-3′), *Sof-dacF1*: (5′-CGGCCAGAAGCACCAGTTAT-3′) and *Sof-dacR1* (5′-CAGTGCTTCACCATTGGGGACT-3′). They were used to amplify respectively a 311, 346, 372, and 400 bp fragment. *Sof-dac* was cloned as described in the protocol of Buresi et al. (2012). Concerning *Sof-six1/2, Sof-eya*, and *Sof-rhodo*psin genes, each of the genes has been synthetized and cloned by Genecust (Dudelange, Luxembourg). The probes for *in situ* hybridization were synthetized from theses plasmids.

### *In situ* hybridization

#### Whole-mount *in situ* hybridization

RNA probes were synthetized with the digoxigenin (DIG) RNA labeling mix from Roche (Mannheim, Germany). According to the sense of PCR product insertion into the vector, sense and antisense probe were obtained with T3 and T7 polymerase (Roche). Spatio-temporal expression patterns of *Sof-six1/2, Sof-eya, Sof-d*ac, and *Sof-opsin* gene transcripts during early embryogenesis of *S. officinalis* (from stage 18 to stage 22) were examined by whole-mount *in situ* hybridization (ISH) according to the protocol detailed in Buresi et al. (2012).

### Cryo-sections *in situ* hybridization

From stage 23 on, it is necessary to observe the expression on sections to localize the expressing cells. Embryos used for cryo-sections *in situ* hybridization were impregnated in 0.12M phosphate buffered (0.08M di-sodium hydrogen ortho-phosphate, 0.02M sodium dihydrogen phosphate dehydrate) plus 30% sucrose treated with 0.1% DEPC (Diethyl pyrocarbonate) for 48 h at 4°C. Then, they were included in Tissue-Tek and blocks were frozen in isopentane cooled at −80°C for 1 min. Sections of 20 μm were performed with cryostat Leica and used for ISH experiments. From stage 23 to stage 30, the expression patterns of those genes were stained by a cryo-sections *in situ* hybridization. Note that control negatives were used for each slide as a test for the same embryo selected and each gene studied. Unless otherwise specified, all steps of the experiments were performed in a humid chamber at room temperature. After 30 min at room temperature, the sections were rehydrated 2 times in 1X phosphate buffered saline (1X PBS) with 0.1% DEPC and treated 1 time in standard 5X saline citrate (75 mM tri-sodium citrate, 0.75 M NaCl) each time for 15 min. A prehybridization step was done in hybridization solution (HS) for 2 h at 65°C (50% deionized formamide, 5X standard saline citrate, 40 μg/ml salmon sperm DNA, 5X Denhardt's, 10% Dextran sulfate). Sections were next incubated overnight at 65°C in HS containing 300 ng/ml of probes. Excess probe was removed by 2 rinses in standard 2X saline citrate (30 mM tri-sodium citrate, 0.3 M NaCl) (respectively 30 min then 1 h, 65°C). Slides were washed for 1 h at 65°C in standard 0.1X saline citrate (0.015 mM tri-sodium citrate, 15 mM NaCl). Sections were then treated twice (15 min each) with MABT (100 mM Maleic acid, 150 mM NaCl, 1% Tween20, pH 7.5). Saturation was performed for 1 h in blocking solution (MABT, 4% Blocking powder (Roche), 15% fetal bovine serum), followed by incubation for 1 h at 4°C with anti-digoxigenin antibodies (Roche) coupled to alkaline phosphatase (AP) and diluted at 1:500 in blocking solution (MABT, 1% Blocking powder, 5% fetal bovine serum). Excess antibody was eliminated by 3 rinses (10 min each) in MABT then 3 rinses (5 min each) in PTW (PBS plus 0.10% Tween20). Sections were impregnated for 20 min in AP solution (100 mM tris hydrochloride, 50 mM Magnesium chloride, 0.1% tween20) with 100 μM levamisole hydrochloride (Sigma, France). The revelation of AP activity was conducted in AP solution (100 mM tris hydrochloride, 50 mM Magnesium chloride, 0.1% tween20 plus 1 mM levamisole hydrochloride) containing 165 μg/ml BCIP (5-bromo-4-chloro-3′-indolyphosphate p-toluidine salt) and 330 μg/ml NBT (nitro-blue tetrazolium chloride) (Roche). The reaction was stopped by washing 3 times (10 min each) in 1X PBS. The slides were treated with DAPI (4′,6-diamidino-2-phenylindole; 25 μg/ml).

For fluorescent *in situ* hybridization, a POD-coupled anti-digoxigenin antibody (Roche) diluted 1:500 was used and bound antibodies were revealed using FITC-tyramide diluted 1:200 in PTW containing 0.001% of hydrogen peroxide, at room temperature for thrice 45 min, in the dark. After washing, the sections were mounted in Mowiol.

### Microscopy observation and image processing

A Leica M16 2F binocular stereomicroscope was used to observe the embryos labeled by *in situ* hybridizations. For cryo-sections ISH, the sections were observed under a Leica DMLB compound microscope. All images were taken by a camera color CoolSnapPro and treated using Adobe Photoshop Elements 9 (Adobe, CA, USA) for contrast and brightness.

## Results

### Eye development and retina differentiation

**Figure 2:**
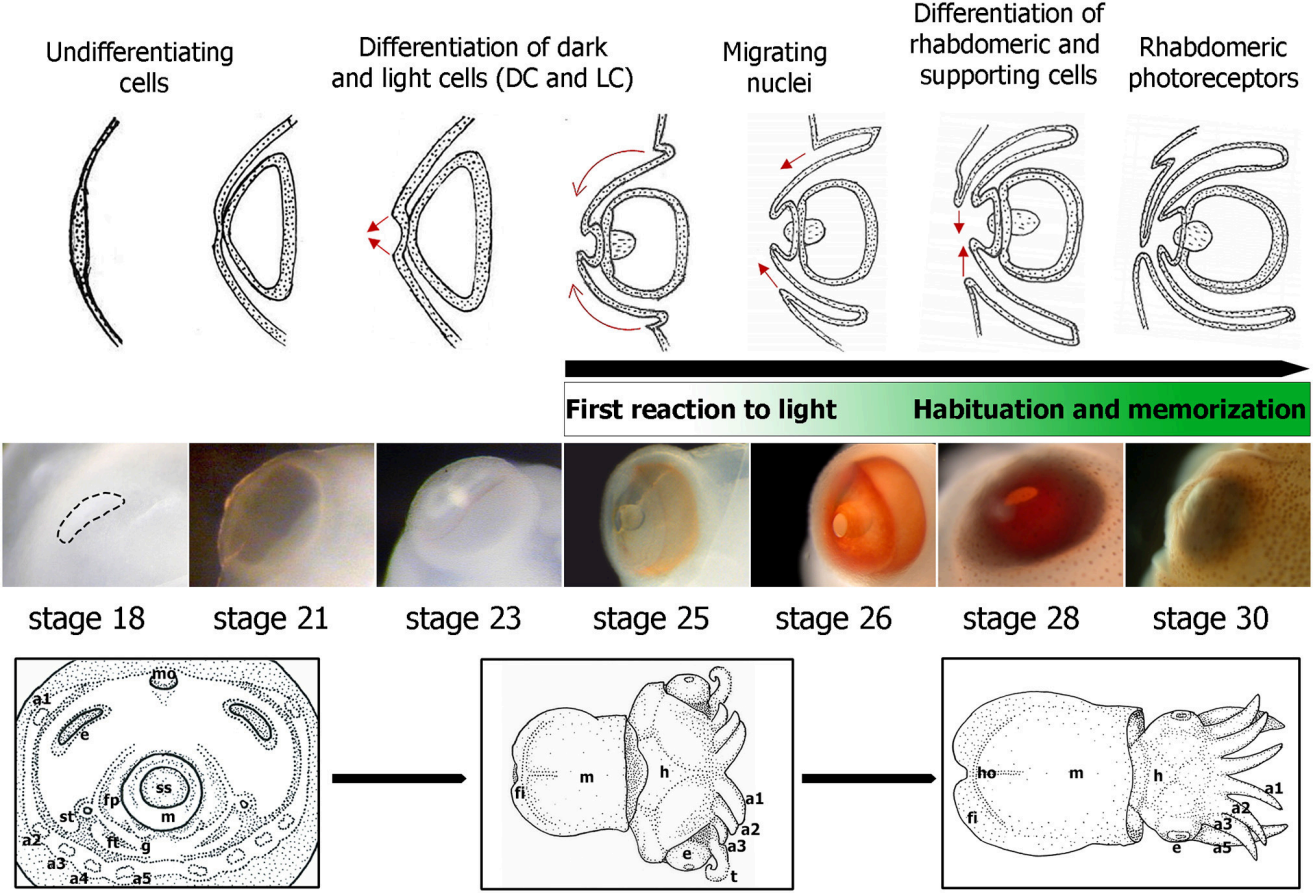
Embryonic eye development in *S. officinalis*. From top to bottom: schematic drawings summarizing the eye morphogenesis between stages 18 and 30 (from Grassé, 1989) with indication of the differentiation of retina cell types based on *Sepiella* (Yamamoto, 1985). Green rectangle: first reaction and habituation to light from stage 25 and the memory process at late stages of development. Eye embryos' photographs between stages 18 and 30. Down: drawings of three major phases of *S. officinalis* organogenesis. a1, a2, a3, a4, a5: arms 1 to 5; e: eye; fi: fin; fp: funnel pouch; ft: funnel tube; g: gill; h: head; ho: hoyle organ; m: mantle; mo: mouth; ss: shell sac; st: statocyste; t: tentacle.

The embryonic development of *S. officinalis* has long been the subject of morphological research (e.g., Naef, 1928; Lemaire, 1971; Boletzky, 1989, 2003; Boletzky et al., 2006; for review Boletzky et al., 2016). The authors have described 30 stages along three main periods. The cephalopod eye is composed of numerous structures, analogous to those of vertebrates (Figure 1) and its development has been described for long time. In *S. japonica*, the development has been described in 40 stages with four phases of retinal differentiation (Yamamoto, 1985). Here we described the development of the *S. officinalis* retina based on that of *S. japonica*. The eye morphogenesis of cuttlefish is characterized by four successive ectodermal folds and begins at stage 15 when the ocular primordium is visible. At stage 16, the invagination of the ocular primordium yields the primary optic vesicle that becomes completely closed at stage 18 (Figure 2). Development of the retina of *S. officinalis* begins at stage 18, when the primary optic vesicle is closed and appears as a single layer with uniform columnar cells (Figure 2). At stage 21, the primary cornea develops from an outer fold surrounding the retinal thickening and the iris develops from an external second ectodermal fold (Figure 2). The lens starts to form at this stage, first teardrop shaped and becomes subspherical at stage 25 (Figure 2). Between stages 21 to 25 (corresponding to stages 24–29 in *S. japonica*), differentiation of two cell types begins. At stage 24, the retina begins to be slightly colored with orange in the periphery of the middle retina. From stage 25 on, the eyes are entirely orange and darken until hatching. Further in the development (stages 25 and 26), a third ectodermal fold covering the eye appears to form a secondary cornea (Figure 2). During the process of establishment of the secondary cornea, the photoreceptor cells and supporting cells begin to differentiate. From stage 28 on, the photoreceptors continue to grow and complete their specific differentiation, forming rhabdomeric cells. The end of the embryonic period at stage 29 is marked by the formation of the eyelid (Figure 2). At hatching, the eye is completely functional (Gilbert et al., 1990).

### Phylogenetic analyses of the *Sof-six1/2, Sof-eya, Sof-dac*, and *Sof-rhodopsin* ESTs

**Figure 3:**
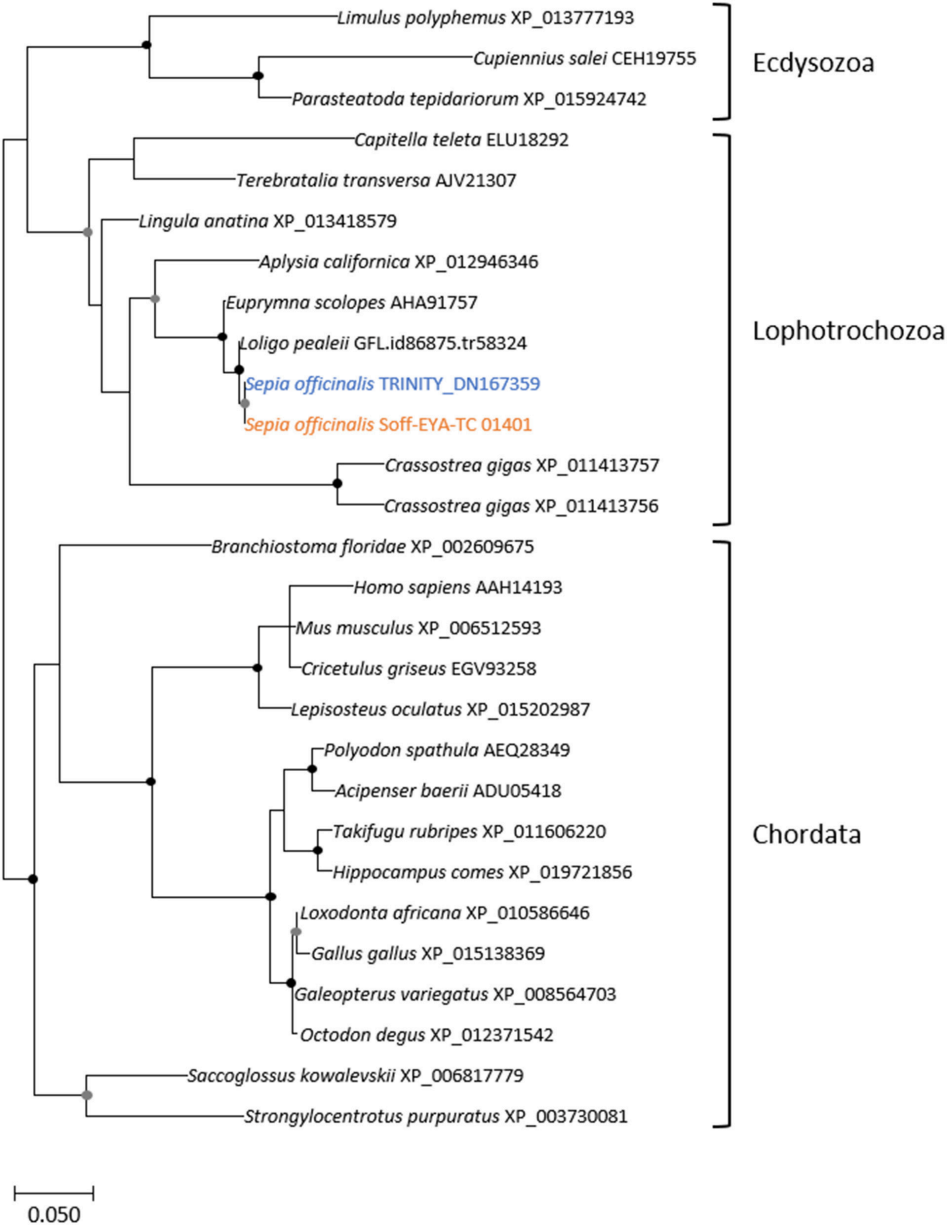
Diversity of eyes absent (EYA) homolog. Maximum-likelihood tree based on 287 aligned amino-acids. The specific *S. officinalis* EST is shown in orange, and the *S. officinalis* contig/gene is in blue. Bootstrap support values are shown in the circles at the nodes (Black, 100–80%; gray, 80–50%).

**Figure 4:**
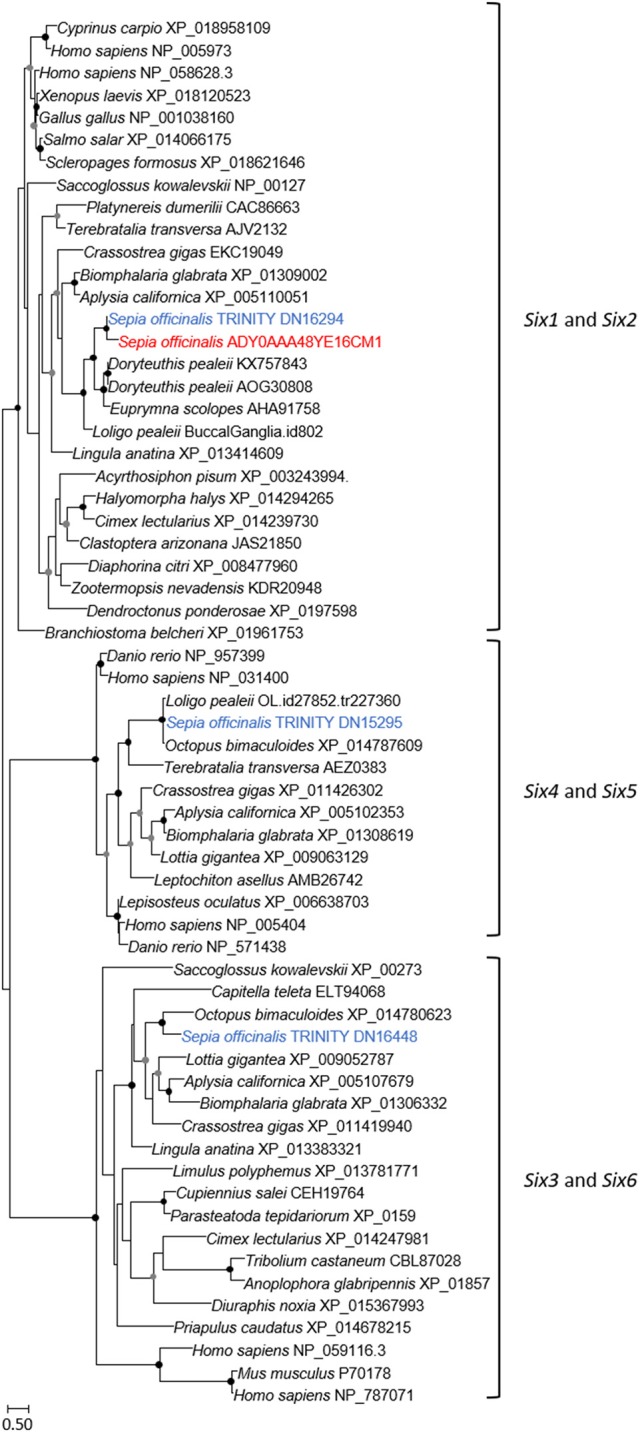
Diversity of sine oculis homeobox (SIX) family. Maximum-likelihood tree based on 183 aligned amino-acids. The specific *S. officinalis* EST is shown in orange, and the *S. officinalis* contigs/genes are shown in blue. For the color code used for the bootstraps refer to Figure 3 legend.

**Figure 5:**
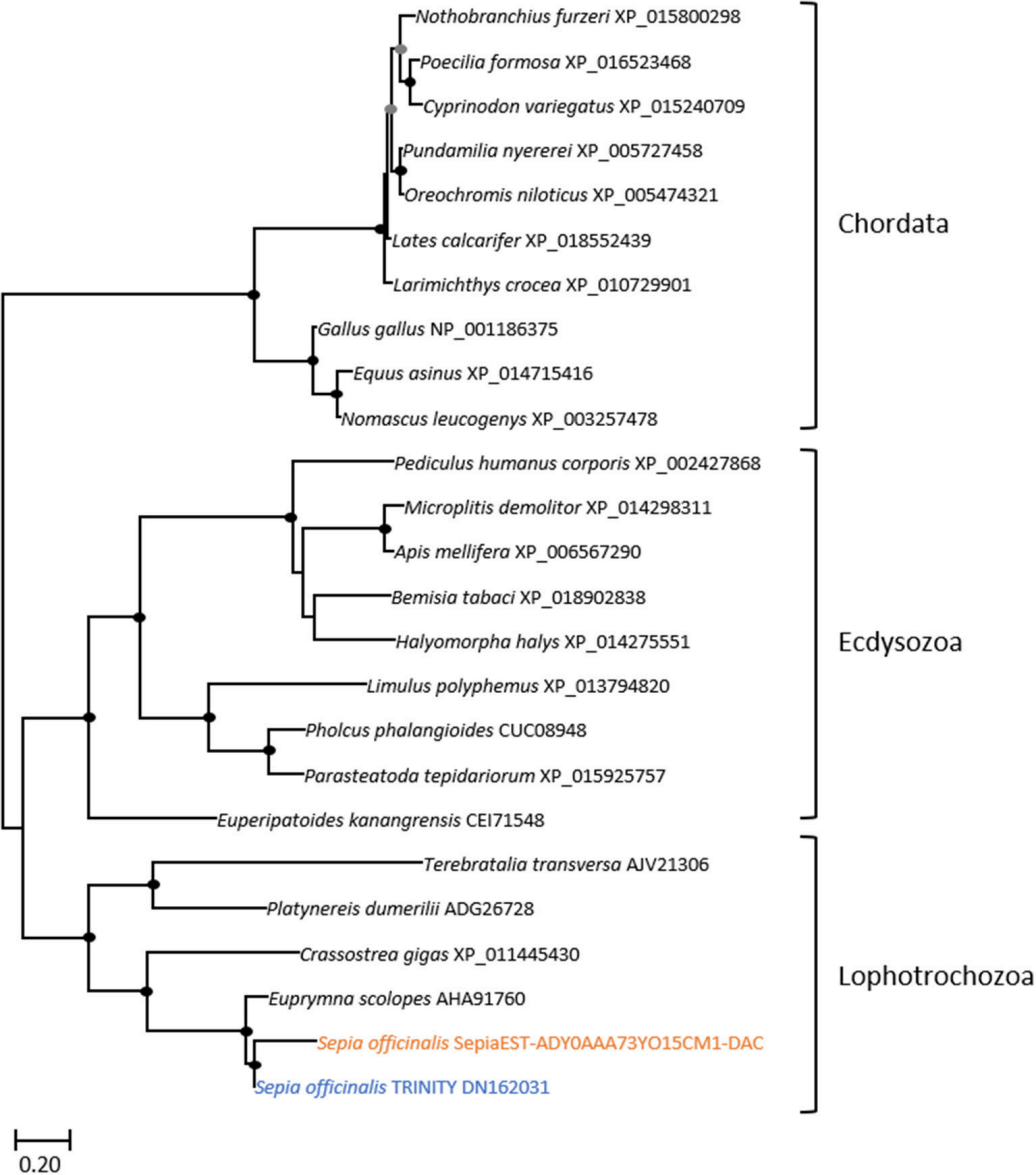
Diversity of dachshund. Maximum-likelihood tree based on 170 aligned amino-acids. The specific *S. officinalis* EST is shown in orange, and the *S. officinalis* contig/gene is shown in blue. The EST and the contig protein sequences are slightly divergent in the N-terminal part of the sequences, probably because of EST sequence errors but importantly only the central and conserved region was used to generate the probe for *in situ* hybridization. For the color code used for the bootstraps refer to Figure 3 legend.

**Figure 6:**
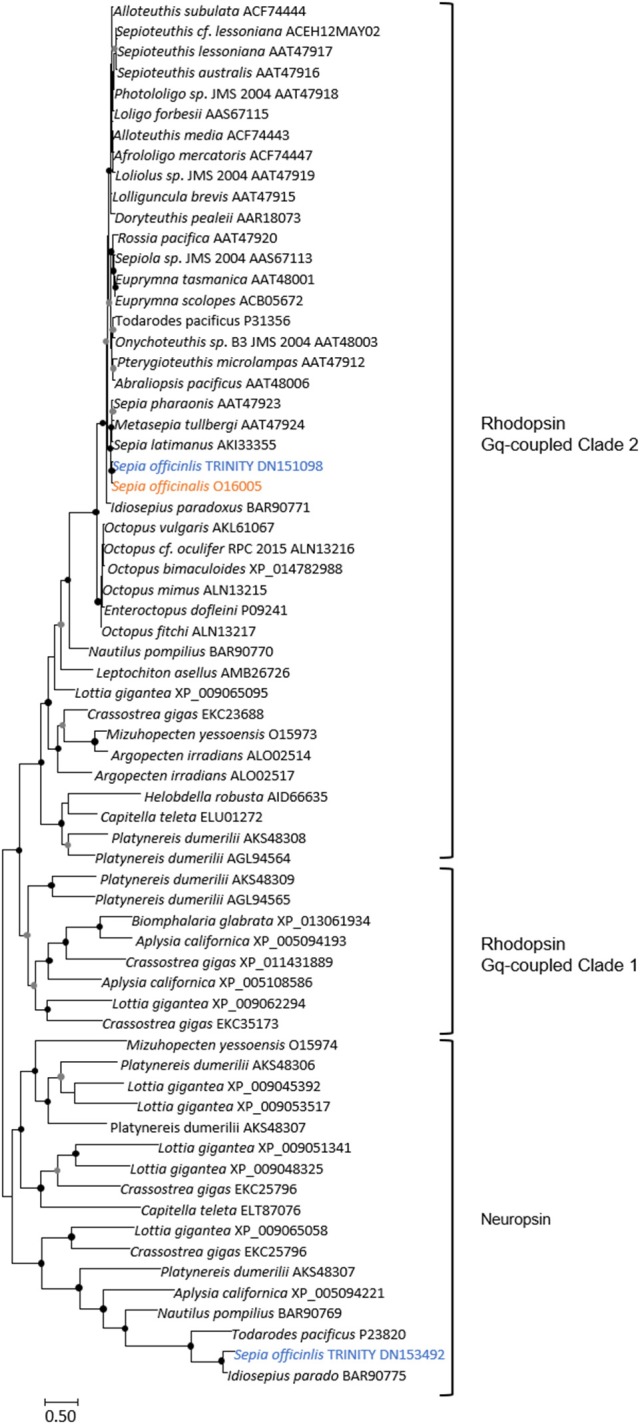
Diversity of *Lophotrochozoa* opsins. This unrooted tree corresponds only to Gq-coupled/rhabdomeric opsins and to Neuropsin types. Maximum-likelihood tree based on 170 aligned amino-acids. The opsin-like protein which identical (100% conserved at the protein level, and 99% (717/718) identities at the nucleotide level); not shown) to the EST used in our study is shown in orange, and the *S. officinalis* contigs/genes are shown in blue. For the color code used for the bootstraps refer to Figure 3 legend.

The eye morphogenesis in numerous metazoans is controlled by several important genes that includes *pax, six, eya*, and *dac*. They were shown to play key developmental roles in non-retinal structures and tissues of vertebrates and *Drosophila*. Blast analyses from our *S. officinalis* EST library revealed the existence of ESTs putatively encoding for *Sof-dac, Sof-six, Sof-eya* and Sof-*opsin*. However, since it was previously shown that cephalopods contain several opsins (Yoshida et al., 2015) or more than one six gene (Koenig et al., 2016), we performed Maximum-likelihood tree based analyses to confirm the identity of ESTs studied here. Using a set of chordate, lophotrochozoan, and ecdysozoan published sequences we obtained a phylogenetic position for the eyes absent (EYA) (Figure 3), sine oculis homeobox (SIX) family (Figure 4), dachshund (Figure 5), and opsins (Figure 6) ESTs. Moreover, to strengthen our phylogenetic analyses, we also used unpublished data corresponding to a recent draft assembly of several transcriptomes of juveniles from *S. officinalis*. Our data demonstrate that our six EST belong to the Six1-Six2 Clade (Figure 4), and that the opsin EST is identical to a previous opsin of the Clade II Gq-coupled/rhabdomeric opsin Yoshida et al., 2015 (Figure 6).

#### *Sof-otx, Sof-six1/2* and *Sof-eya* expressions in the developing eyes of *Sepia officinalis*

**Figure 7:**
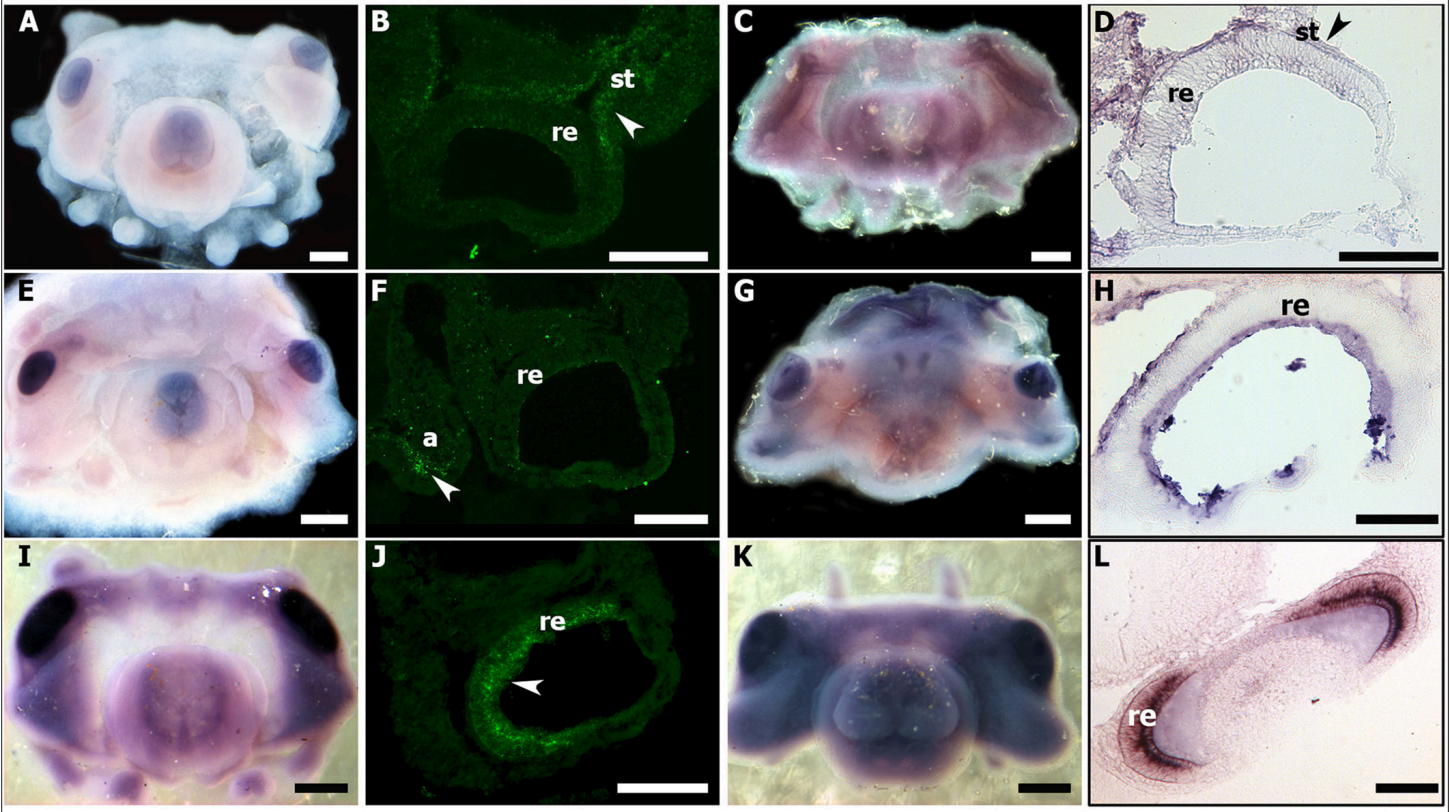
*Sof-eya, Sof-dac*, and *Sof-otx* expressions during stage 20 and 21 of *S. officinalis*. **(A–D)**: *Sof-eya*. **(E–H)**: *Sof-dac*. **(I–L)**: *Sof-otx*. **(A,E,I)**: embryos stage 20. **(B,F,J)**: thin sections of stage 20. White head arrows point expressions of genes. **(C,G,K)**: embryos stage 21. The pink color in eyes and around the eyes is background: no staining is visible through sections **(D,H,L)** [thin sections of embryos **(C,G,K)**, respectively]. a: arms; re: retina; st: surrounding tissue. **(A,C,E,G,I,K)**: scale bar is 500 μm. **(B,D,F,H,J,L)**: scale bar is 150 μm.

In the whole-mount embryo, *Sof-eya* transcript appears expressed at stages 20 and 21 in the eye area (Figures 7A,C). *In situ* hybridization on cryo-sections, allowing a more precise tissue localization shows a staining localized only in the tissue surrounding the eyes at stage 20 and 21 (Figures 7B–D). In contrast, *Sof-six1/2* expression is not detected in the surrounding tissue (data not shown). For both genes (*Sof-six1/2* and *Sof-eya)*, no expression is observed in the retina during these stages and from stage 23 to hatching, no expression is detected in any other tissues. By contrast, *Sof-otx* is expressed in the eye area as shown in whole-mount embryo ISH and this expression is located precisely in the retina at stage 20 and 21 (Figures 7I–L). The expression in the retina stops after stage 26 (Buresi et al., 2012).

#### *Sof-dac* expression during organogenesis

**Figure 8:**
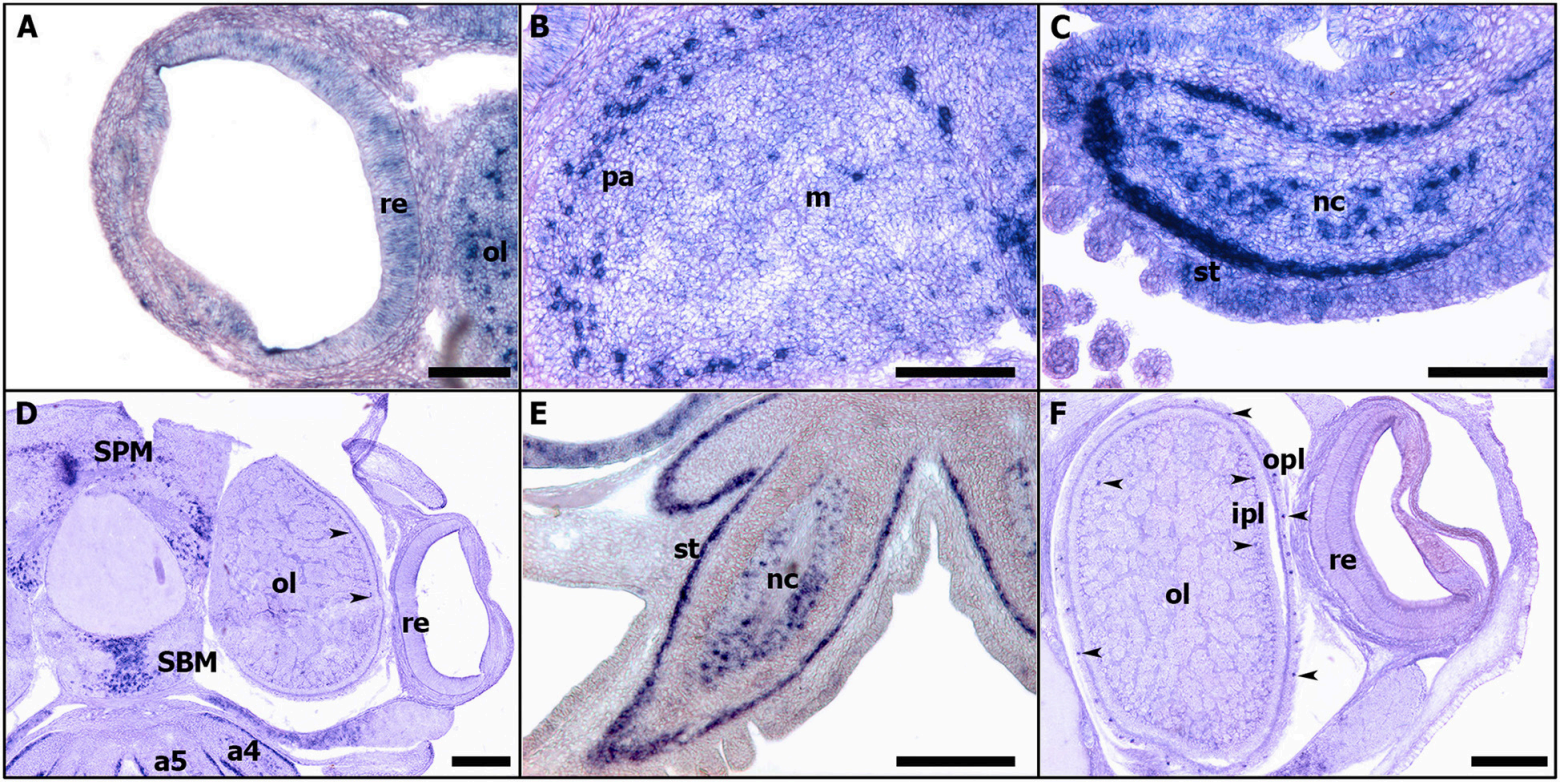
Expressions of *Sof-dac* during *S. officinalis* organogenesis on transversal cryo-sections. Dorsal side is up and ventral side is down. **(A–C)**: stage 24. **(A)**: eye. **(B)**: optic lobe. **(C)**: arm 5. **(D,E)**: stage 26. **(D)**: expressions in the supraesophageal and subesophageal masses, in inner and outer plexiform layers (black head arrow) and medullar zone of the optic lobes. **(E)**: arm 5. **(F)**: stage 28, optic lobe and eye. Black head arrows point out the expressions of the respective genes in the eyes. a4, a5: arms; ipl: inner plexiform layer; m: medulla; nc: nerve cord; ol: optic lobes; opl: outer plexiform layer; pa: plexiform area; re: retina; SBM: subesophageal mass; SPM: supraesophageal mass; st: surrounding tissue. **(A–C)**: scale bar is 150 μm. **(D–F)**: scale bar is 300 μm.

In all studied stages (from 18 to 30), *Sof-dac* appears expressed in the eye area. *Sof-dac* expression begins at stage 18 when the primary optic vesicles are not yet closed. The expression is observed in the eye area, in tissues surrounding the primary optic vesicle and in peripheral structures such as the arms (Figures 7E–H). By ISH on sections, allowing tissue localization, we show that there is expression neither in the retina nor in other tissues surrounding the eyes at early organogenesis (Figures 7F–H). No expression of *Sof-dac* is observed in the retina in later stages, from 24 to hatching. Nevertheless, *Sof-dac* is expressed, in the arms, cerebroid ganglia, visceral ganglia, and gills (Figure 8). *Sof-dac* expression is maintained in the arms until late stages of development (Figures 8C,E). At stage 24, when the ganglia merge and begin to differentiate into lobes, *in situ* hybridization on cryo-sections shows that *Sof-dac* expression is located in the plexiform area and medullar zone of the optic lobes, in some cells of the arm cord, in tissue layer surrounding the nerve cord, supraesophageal (SPM) and subesophageal (SBM) masses (Figures 8B,C). From stage 25 to 30, *Sof-dac* expression is restricted to the developing central nervous system, especially SPM and SBM, in some cells of the arm cord and in tissue layer surrounding the nerve cord, in the inner and outer plexiform layers and central medulla of the optic lobes (Figures 8D–F).

#### *Sof-rhodopsin* expression in the differentiating retina

**Figure 9:**
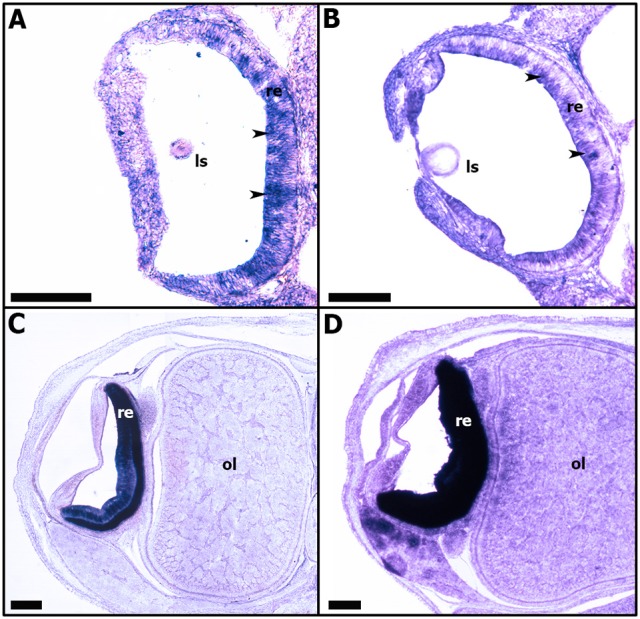
Expressions of *Sof-rhodopsin* during *S. officinalis* organogenesis on transversal cryo-sections. Dorsal side is up and ventral side is down. **(A,B)**: expressions in the developing retina (black head arrows) respectively at stages 23 and 25. **(C,D)**: expressions in differentiating retina, respectively at stages 28 and 30. ls: lens; ol: optic lobe; re: retina. Scale bar is 200 μm.

From stage 23, *Sof-rhodopsin* expression is detected only in the undifferentiated retina (Figure 9A). *Sof-rhodopsin* expression in the retina continued through stage 30 before hatching (Figure 9). This expression is variable; from stage 23 to 25, *Sof-rhodopsin* is expressed weakly and is observed only in the outer portion of the retina, corresponding to the area where the nuclei of the undifferentiated receptor cells and support cells are localized (Figures 9A,B). From stages 28 to 30, *Sof-rhodopsin* appears strongly expressed in the entire retina (Figures 9C,D). Actually, *rhodopsin* is normally restricted to the photoreceptor cells and is not present in all cells. Our observation is probably due to the numerous and juxtaposed rhabdomeric photoreceptors at the end of the development. *Sof-rhodopsin* expression should be limited to the outer segments of the differentiated retina cells.

## Discussion

### Eye specification gene expression in *S. officinalis* embryo

Our expression data show that *Sof-eya* expression is restricted to the eye area and surrounding tissues at stages 20 and 21. *Sof-six1/2* is also expressed in the eye area during the early stages of organogenesis (from stage 20 to stage 21) in *S. officinalis*. Surprisingly, no *Sof-six1/2* and *Sof-eya* expression is shown in the eye area before stage 20 and after stage 22. *Eya* and Six have been strongly proposed to have an ancient role in eye development and their orthologs are involved in visual system development in both “invertebrates” and vertebrates (Vopalensky and Kozmik, 2009). The analysis of *Eya* genes expression in vertebrate eye development has shown that the three *Eya* genes, *Eya1, 2, 3* are differentially expressed in the developing eye (Xu and Saunders, 1997). Indeed, *Eya1* is expressed in the lens, optic stalk, and neural retina (Xu et al., 1997). *Eya2* is expressed in the neural retina only. Eya3 is present in the optic vesicle and the periocular mesenchyme, but both genes Eya2 and Eya3 are absent in the lens (Xu et al., 1997). In *Drosophila, sine oculis* has been shown to be required for the development of both, the compound eyes and the ocelli (Cheyette et al., 1994). Indeed, it has a critical role for the development of the entire visual system (Gehring, 2002). In mouse, *sine oculis* orthologs *six1/six2* are known to be expressed in the adult differentiating cells of the retina (Oliver et al., 1995). In protostome, *six1/2* homologs are known to be important for the early specification of the visual system (Cheyette et al., 1994; Arendt et al., 2002). The role of *six1/2* homologs in early visual system specification has been proposed to be evolutionary conserved outside Bilateria (Stierwald et al., 2004). Recent studies in cephalopods indicate that *six2, six3*, and *eya* expressions have been observed in the eye area, the optic lobes and often, at very early stages: in the lip of the placode and edges of the lid in *D. pealeii* embryo until stage 27. This suggests that six genes and *eya* are involved in lens formation as it is observed in vertebrates (Koenig et al., 2016). In *E. scolopes, six* and *eya* expressions were detected in numerous tissues including, until stage 26, the ventral light organ, with no known “visual function” (Peyer et al., 2014). However, in the results shown in these studies, expressions are presented by whole-mount ISH without showing precisely the staining in the eye fields with convincing histological data. According to our results both on sections and whole-mount, we point out that the expression shown in whole mount ISH in these studies must be confirmed by sections in the areas supposed to be stained. Furthermore, Ogura et al. (2013) have shown that *six3/6* is involved in the lens formation and its expression is localized in the lentigenic cells. Studies from vertebrates and *Drosophila* and even cnidarian, report *six3/6* expression in the developing eye (Oliver et al., 1995; Loosli et al., 1999; Zuber et al., 1999). Although we encountered problems and were not able to repeat staining of *six1/2* expression on sections, our expression data do not suggest any evolutionary conserved role of *Sof-six1/2* in the retinal specification and the eye formation. However, we cannot exclude the expression of another *six*-subfamily gene such as *six3/6* proposed to have an evolutionary conserved role. *Sof-dac* transcript is expressed from stage 18 to stage 30 in eyes and the optic lobes (Figure 8) but surprisingly its expression is never observed in the retina of cuttlefish. In vertebrates and *Drosophila, dac* expression is observed in the central nervous system, optic cup and also in the entire neural retina as it is shown on sections (Hammond et al., 1998; Caubit et al., 1999; Heanue et al., 1999; Loosli et al., 2002; Martín-Durán et al., 2012). In molluscs, *dac* is expressed in eye photoreceptor cell's development in larval chiton (Vöcking et al., 2015). In a cephalopod, *E. scolopes, dac* transcript expression is observed in the eyes, arms, mantle and light organs by whole mount *in situ* hybridization (Peyer et al., 2014). Strikingly, this expression is shown only on sections in the light organs but not in the retina. We point out that *dac* expression at the cellular level of eyes and other tissues must be confirmed by sections. In addition to being expressed in eye-associated tissues, *Sof-dac* has been also stained in supra-esophageal and sub-esophageal masses, gills, arms, and statocysts, all tissues playing important roles in sensory-motors functions. Our results show that the role of *dac* transcript is conserved in the central nervous system of metazoans. Nevertheless, *Sof-dac* seems expressed in optic lobes, precisely in the inner, the outer plexiform layers and also in the medullar zone at late stages until stage 30 before hatching. The plexiform area is also called the “deep retina” due to its similarity with the ganglionic layer of the vertebrate retina (Young, 1962). It is constituted by an inner and outer plexiform layer separated by a complex neuropil zone (Young, 1974). In cephalopods, the plexiform area contains several cell types (amacrine neurons, centrifugal, centrally, and centripetal cells). Furthermore, the retinal photoreceptors connect with the optic lobes by the photoreceptor axons in the plexiform area (Hartline and Lange, 1974). Indeed, the photoreceptors make synaptic contact with the amacrine neurons located in the inner and outer plexiform layers (Young, 1971; Case et al., 1972). *Sof-dac* expression in these areas could be an indication of the presence of photoreceptive cells in the optic lobes. These results suggest that *Sof-dac* is involved in the morphogenesis of the eye and visual control structures such as optic lobes in developing *S. officinalis*. In addition, we evidenced unexpected *Sof-dac* expression, for the first time: in some cells of the arm cord and in tissue layer surrounding the nerve cord. The histological character of this tissue is not yet identified, and additional studies must be done to determine if it is nervous or muscular cells as shown in some species (Heanue et al., 1999).

In *S. officinalis* we evidence that the gene network, *six1/2, eya*, and *dac* is involved in the early eye area formation but not in the differentiation of the retina. Similar results were found with *Pax6*, not involved in the retina formation (Navet et al., 2017). *Pax6* is considered as a universal master control gene of eye formation and developing photoreceptors in metazoans (Gehring, 2005; see review in Kumar, 2009); it regulates upstream the RDGN, which instructs the formation of the adult eye in *Drosophila*. In numerous cephalopods (*L. opalescens*: Tomarev et al., 1997; *E. scolopes*: Hartmann et al., 2003; Peyer et al., 2014; *S. officinalis*: Navet et al., 2009, 2017; *I. paradoxus*: Yoshida et al., 2014; *D. pealeii*: Koenig et al., 2016), *Pax6* expression has been shown in optical areas, eye and optic lobes. In *S. officinalis, Pax6* presents a large ectodermic and mesodermic expression in the optic area but never in the retina (Buresi et al., 2016; Navet et al., 2017) and no clear expression in retina cells has been finally shown in the other species. These findings suggest the non-conservation of *pax6* in the differentiation of the retinal photoreceptors by the loss of the conserved RDGN upstream regulation: this could explain the loss of expression of *six, eya*, and *dac* in the retina. No other *pax* genes (*pax3/7* and *pax2/5/8*) is expressed in *S. officinalis* embryo all along the development in the retina cells from the first steps of specification until the rhabdomeric photoreceptors are fully differentiated. Nevertheless, other genes appears to have a role in retina differentiation, such as *Sof-otx*, which is expressed in the retina as already shown (Buresi et al., 2012).

Indeed, *otx2* transcript factor is known to play a role equally in the development of the eye and the photoreceptor cells. In vertebrates, *otx* orthologs could control all retinal cell types (Chen et al., 1997; Viczian et al., 2003). *Otx2* and *pax6* are expressed upstream of the opsin: they could act to enhance r-opsin expression for the rhabdomeric photoreceptor; but only *otx2* (and not *pax6*) with Rx activate the expression of c-opsin, characteristic of ciliary photoreceptors (Vopalensky and Kozmik, 2009). *Otx2* transcription factor expression is reported in the photoreceptors of the fly ommatidia, in the photoreceptor precursors of larval eyes of *T. transversa* and the planarian pigment-cup eyes (Umesono et al., 1999; Passamaneck et al., 2011). In *Sepia* embryos, *otx* is expressed in the eyes particularly in the retina from early to late developmental stages (from stage 19 to stage 26) when the photoreceptor's differentiation started but its expression is not found from stage 26 when the retinal cell type organization is being achieved (Buresi et al., 2012). Thus, the mystery of the final differentiation of retinal photoreceptors and the genes that control this process remains to be characterized in order to understand what the underlying molecular mechanisms are.

### *Sof-rhodopsin* expression in the differentiating rhabdomeric photoreceptors

Our results reveal for the first time in *S. officinalis* embryo the expression patterns of *rhodopsin* transcript in the developing retina from stage 23 to hatching (Figure 9). Several cephalopod species have both retinal and extraocular photoreceptors located in the light organ, skin, paraolfactory vesicles, epistellar body, and nervous system where a great diversity of *opsin* proteins can be found (Cobb and Williamson, 1998; Tong et al., 2009). In coleoid cephalopods, the retina only has a single layer containing rhabdomeric photoreceptors, supporting cells and blood vessels (Messenger, 1981). Additionally, the retinal rhabdomeric photoreceptors are known to express a single type of *rhodopsin* in coleoid cephalopods (Bellingham et al., 1998). *Rhodopsin* transcript has been evidenced by RT-PCR and immunolabeling not only in the retinas of several adult species of cephalopods such as squid *D. pealeii*, cuttlefish *S. officinalis* and *Sepia latimanus* but also in the skin of the same species (Kingston et al., 2015a,b). These authors indicate that the *rhodopsin* detected both in the skin and retina of adult cuttlefish is the same. But interestingly, in *S. officinalis* embryo, unlike adults, we have evidenced *rhodopsin* expression only in the retina, not in other tissues or structures, such as the skin. As the *rhodopsin* sequence used is exactly the same described in Kingston et al. (2015a) and Yoshida et al. (2015), and as no other *rhodopsin* has been found in our EST-library, we propose that *rhodopsin* is expressed in the skin after hatching, in the juveniles, when the patterns are useful for camouflage. A differential expression of rhodopsin during development has been shown in annelid suggesting a control of the “visual function” in accordance with developmental stage (Randel et al., 2013). Finally, *rhodopsin* transcript can be expressed so weakly that it cannot be detected by *in situ* hybridization. The weak expression of *Sof-rhodopsin* observed between stages 23 and 25 can be explained by the first differentiation step of cells at stage 23, and the beginning of the differentiation into photoreceptor cells and supporting cells from stage 25 on, as observed in *Sepiella*. This expression might be localized in the cells which differentiate into rhabdomeric photoreceptors at late stages as it is observed in other cephalopod adult species. But, in order to link *sof-rhodopsin* expression to the retinal cell differentiation process, further studies should be conducted during cuttlefish embryogenesis. Moreover, the expression of *Sof-otx* in cuttlefish's retina at stage 19, long before the expression of *rhodopsin* (stage 23) is congruent with the upstream regulation of *rhodopsin* in vertebrates (Vopalensky and Kozmik, 2009), and the timing of retina cone differentiation in mouse preceding the *rhodopsin* expression (Rodgers et al., 2016). Nevertheless, as *Sof-otx* expression stopped at stage 26 (Buresi et al., 2012), we question the control of *rhodopsin* expression in late developmental stages, i.e., in rhabdomeric photoreceptors. This expression and the production of *visual rhodopsin* combined with the observation of reactivity of the eye from stage 25 on show that *rhodopsin* is present before the setting up of the rhabdomeric photoreceptors (Yamamoto et al., 1985). These data strengthen the hypothesis of Romagny et al. (2012) suggesting that *S. officinalis* is able to react to light stimuli from stage 25 of organogenesis on, when the first retinal pigments appear, a stage when the rhabdomes are not totally differentiated. As a consequence, a fully differentiated rhabdomeric photoreceptor is not necessary to have a “basic” answer to light stimulus. Nevertheless, Romagny et al. (2012) have shown, by behavioral experiments of answer to light, that the habituation to light, the memory process is evidenced only at late stages of development, when the retinal rhabdomeric photoreceptors are totally differentiated and when the *rhodopsin* expression is very high (Figure 9). It is linked to the final maturation of the analysis centers (brain and optic lobes).

## Conclusion

The aim of this study was to lay the molecular basis of eye formation, differentiation, and specification of retinal photoreceptors in *S. officinalis*. The results obtained indicate that three genes important for eye morphogenesis and photoreceptors differentiation in numerous groups of metazoans are involved in eye formation but never in retina cell differentiation during *S. officinalis* embryonic stages studied (17 to 30). We cannot exclude that they are expressed before stage 17 but as there are undifferentiated cells until stage 21, their role in the retina cell differentiation would be questioned. These findings reveal or reinforce the divergence and the broad complexity in the genetic network underlying the cephalopod retinal differentiation. *Sof-six1/2, Sof*-*eya*, and *Sof-dac* are localized only in the eye area questioning about the gene network involved in the differentiation of rhabdomeric photoreceptors in cephalopods. Actually, all of the previous studies in eye development in cephalopods have been performed by whole mount *in situ* hybridization. However, according to our results, we point out that gene expressions must be studied on sections to make it possible to exactly locate the expression of these genes at the cellular level. Nevertheless, cephalopods are an interesting model to study the evolution of the nervous system, the eye development complexity and the diversity of photosensitive structures. Besides the fact that RDGN expression levels could be outside the threshold of detection, the *Sepia's* retina development and the final differentiation of rhabdomeric photoreceptors is probably controlled by other genes than those identified until now. Thus, it will be necessary to explore the role of other genes such as *Notch* that is known to intervene in the retina and lens formation in vertebrates and *Drosophila* where it regulates the cell cycle progression within retina and lens (Livesey and Cepko, 2001; Charlton-Perkins et al., 2011).

Our study opens up other opportunities to investigate the evolution of functions complexity within metazoans. *Sof-rhodopsin* expression in the retina during *S. officinalis* embryogenesis correlates with the behavioral observation and the light sensitivity of cuttlefish embryos before the final differentiation of rhabdomes. Nevertheless, it seems necessary to investigate the diversity of photoreception molecules, the character of tissues and cells that expressed these molecules, in skin, optic lobes, and brain, to build an understanding about the evolution of photosensitive structures and phototransduction function in the retina and in the extraocular photoreceptor tissues. As it is described above, other genes involved in the phototransduction cascade and/or signaling pathways must be explored such as *arrestin* already identified in *E. scolopes* adult eye (Tong et al., 2009).

## Ethics statement

We use cephalopod embryos before hatching. In this case, according to the European law, our protocol is not in the field of animal experiment and there is no need to obtain an authorization from our local committee (MNHN Commitee Cuvier N) which has been consulted. LB-P is a member of Cuvier Commitee.

## Author contributions

Conceived of project: LB-P; Designed experiments: LB-P, BI, and AA; Executed experiments: BI and AA; Analyzed data: BI; Phylogenetic analysis: PL; Edited manuscript: BI, LB-P, PL, AA and YB.

### Conflict of interest statement

The authors declare that the research was conducted in the absence of any commercial or financial relationships that could be construed as a potential conflict of interest.
